# Breaking Cycles of Trauma and Addiction in Youth under Educational Custody: Contributions of Psychology and Psychiatry through Mutual Aid Groups

**DOI:** 10.1192/j.eurpsy.2026.11167

**Published:** 2026-07-31

**Authors:** M. A. da Fonseca, F. Magalhães

**Affiliations:** IDIS, Vila Nova de Gaia, Portugal

## Abstract

**Introduction:**

Adolescents in juvenile custodial settings often experience complex trauma, psychiatric disorders and substance use, which collectively hinder rehabilitation and increase maladaptive behaviors. This study aims to investigate the role of Mutual Aid Groups (MAGs), integrating psychological and psychiatric approaches, in promoting trauma-informed rehabilitation. A mixed-methods design will be conducted with N = [8] adolescents, at the time under custodial measures.Pre- and post-intervention evaluations are expected to validate reductions in trauma-related and depressive symptoms, decreased substance use patterns and improved psychosocial functioning.

**Objectives:**

To evaluate the effectiveness of Mutual Aid Groups (MAGs), integrating psychological and psychiatric approaches, in promoting trauma-informed rehabilitation among adolescents in juvenile custodial settings; To assess the baseline levels of trauma-related symptoms, depression and substance use in adolescents under custodial measures; To implement and monitor a 10-week Mutual Aid Group intervention focused on peer support, emotional regulation and adaptive coping strategies; To analyze the contribution of interdisciplinary collaboration—between psychologists and child and adolescent psychiatrists—in enhancing therapeutic engagement

**Methods:**

**Procedures**

At baseline (T0), all eligible participants will complete a battery of standardized self-report and clinician-administered measures assessing trauma-related symptoms, depressive symptoms, substance use and psychosocial functioning. Participants will attended the **10-week MAG intervention**, delivered in weekly 90-minute sessions. Post-intervention assessments (T1) will be conducted within two weeks after program completion.

**Results:**

Based on the present study design and objectives, the following results could potentially emerge: **Reduction in trauma-related and depressive symptoms, Decrease in substance use patterns, Improvement in psychosocial functioning, Role of interdisciplinary collaboration**

**Conclusions:**

This study is expected to support the effectiveness of Mutual Aid Groups (MAGs), integrated with psychological and psychiatric approaches, as a trauma-informed intervention model for adolescents in juvenile custodial settings. The anticipated findings point toward a significant reduction in trauma-related and depressive symptoms, a decline in substance use behaviors, and improvements in emotional regulation and psychosocial functioning. Furthermore, the interdisciplinary collaboration between psychologists and child and adolescent psychiatrists is expected to emerge as a critical factor in enhancing therapeutic engagement, ensuring clinical safety, and customizing interventions to the complex needs of this high-risk population.

**Disclosure of Interest:**

None Declared

